# Prevalence of Chinook salmon is higher for southern than for northern resident killer whales in summer hot-spot feeding areas

**DOI:** 10.1371/journal.pone.0311388

**Published:** 2024-10-10

**Authors:** Burak Saygili, Andrew W. Trites

**Affiliations:** Marine Mammal Research Unit, Institute for the Oceans and Fisheries, The University of British Columbia, Vancouver, BC, Canada; National Cheng Kung University, TAIWAN

## Abstract

Differences in the availability of prey may explain the low numbers of southern resident killer whales and the increase in northern resident killer whales in British Columbia and Washington State. However, in-situ data on the availability of their preferred prey (Chinook salmon, *Oncorhynchus tshawytscha*) in the core feeding areas used by these two populations of fish-eating killer whales have been lacking to test this hypothesis. We used multi-frequency echosounders (38, 70, 120, and 200 kHz) to estimate densities of adult Chinook (age-4+, > 81 cm) within 16 hot-spot feeding areas used by resident killer whales during summer 2020 in the Salish Sea and North Island Waters. We found Chinook were generally concentrated within 50 m from the bottom in the deep waters, and tended to be absent near the surface in the shallow waters (< 50 m). In general, the densities of Chinook we encountered were highest as the fish entered the Salish Sea (from Swiftsure Bank in the south) and Johnstone Strait (from Queen Charlotte Strait to the north)—and declined as fish migrated eastward along the shoreline of Vancouver Island. Median densities of Chinook for all sampled areas combined were 0.4 ind.·1000 m^−2^ in northern resident foraging areas, and 0.9 ind.·1000 m^−2^ in southern resident killer whale areas (*p* < 0.05, Mann–Whitney *U* test). Thus, Chinook salmon were twice as prevalent within the hot-spot feeding areas of southern versus northern resident killer whales. This implies that southern resident killer whales have greater access to Chinook salmon compared to northern residents during summer—and that any food shortage southern residents may be encountering is occurring at other times of year, or elsewhere in their range.

## Introduction

Two populations of fish-eating killer whales (*Orcinus orca*) frequent the coastal waters of British Columbia where they preferentially consume Chinook salmon (*Oncorhynchus tshawytscha*) [1–5]. One of these populations, northern resident killer whales, ranges from southern British Columbia to Southeast Alaska—and has tripled to over 300 individuals since monitoring began in the early 1970s [6]. In contrast, the southern resident killer whale population, which inhabits the waters between southern British Columbia and California, has not experienced any sustained growth during this time. They have fluctuated between 66 and 98 individuals, and numbered 74 as of December 2023 [7]. The differing trajectories of these two populations of fish-eating killer whales have been attributed to ecological and biological differences between regions such as prey availability, diet breadth, competition, physical disturbance, underwater noise, contaminants and inbreeding [8–13]. However, food availability likely plays the greatest role in limiting their carrying capacities.

Positive correlations have been reported between broad-scale indices of Chinook abundance and southern resident killer whale demographics [12, 14–16]. However, attempts to link the correlations to underlying causal mechanisms have failed to identify specific Chinook stocks of importance, and have not been able to determine when or where reduced Chinook abundance might be affecting southern resident killer whale numbers [16]. The correlations have also weakened as additional years of data have been analyzed [17]—and have similarly failed to provide insights into the spatial and temporal scale of overlaps between predators and prey required to manage fisheries and mitigate negative impacts of human activities on killer whales.

An alternative to using statistical indices of abundance to assess the hypothesis that fewer Chinook are available to southern resident killer whales than to northern resident killer whales is to directly measure the distribution and density of Chinook salmon in the habitats used by the two populations of killer whales. Numbers of fish, sizes of schools, body sizes of individuals, and depths of occurrences can be determined in real time using ship-based echosounders that collect high-resolution data with extensive spatial coverage through the water column [5]. Acoustics can also provide measures of relative density and abundance to estimate the sizes of individual fish, school densities and overall abundances [18–20].

In 2018 and 2019, Sato et al. [5] completed broad scale acoustic surveys during summer (July and August) of Juan de Fuca Strait (where southern resident killer whales feed) and Johnstone Strait (where northern resident killer whales feed). They found that both killer whale habitats had patchy distributions of Chinook that did not differ in the size of fish or their frequency of occurrence. However, densities of fish were 4–6 times higher in Juan de Fuca Strait, which countered the hypothesis that fewer prey were available to southern resident killer whales.

**Fig 1:**
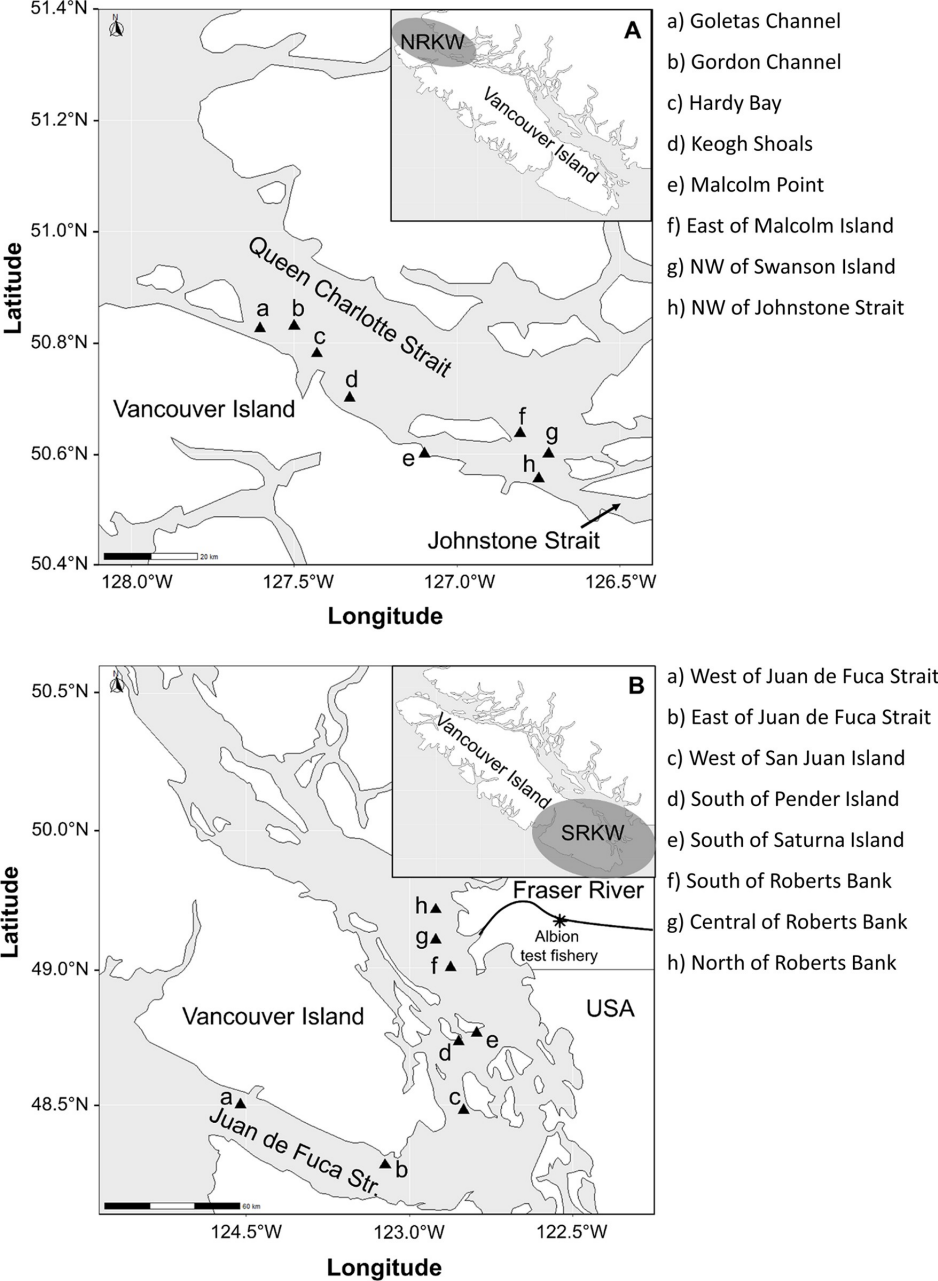
Sampling locations within the Northeast Pacific Ocean. A) The 8 hot-spot feeding areas sampled within a portion of the northern resident killer whale (NRKW) habitat ranging from Queen Charlotte Strait to Johnstone Strait. B) The locations of 8 sampled feeding areas within a portion of southern resident killer whale (SRKW) habitat ranging from the entrance of Juan de Fuca Strait to the mouth of the Fraser River. Base maps were drawn using the R packages rnaturalearth and sf with free vector and raster map data from naturalearthdata.com.

In 2020, we expanded the prey sampling areas previously assessed with hydroacoustics to include greater portions of the summer feeding areas used by resident killer whales in the Salish Sea (from the entrance of Juan de Fuca Strait to the mouth of the Fraser River) and in the North Island Waters (from Queen Charlotte Strait to Johnstone Strait) (Fig 1). Within each region, we collected acoustic data on finer spatial scales from 16 hot-spot feeding areas frequented by resident killer whales and sport fisheries. Meetings and workshops held with whale watching companies and professional sport fishers were used to identify the hot-spot areas on nautical charts where whales were most frequently observed feeding, and where concentrations of fish targeted by fisheries were highest. Thus, we collected in-situ data on the presence of prey to test whether the availability of Chinook in hot-spot feeding areas was lower in the Salish Sea compared to the North Island Waters during late summer—consistent with the hypothesis that fewer Chinook salmon are available to southern resident killer whales than to northern resident killer whales.

## Material and methods

### Study site, survey design and data collection

We consulted sport fishing guides and owners of whale watching companies to identify areas where Chinook are known to concentrate and killer whales are known to feed. Based on their collective knowledge, we identified 8 hot-spot feeding areas in the Salish Sea (from the entrance of Juan de Fuca Strait to the mouth of the Fraser River) and 8 in the North Island Waters (from Queen Charlotte Strait to Johnstone Strait) (Fig 1). All areas were considered nearshore (i.e., within a median distance of 700 m).

Our surveys consisted of zigzagged line transects (86 acoustic transects in the Salish Sea, and 101 transects in the North Island Waters) that encompassed a broader geographic region than assessed by Sato et al. [5] in Juan de Fuca Strait and Johnstone Strait in 2018 and 2019—and were designed to compare and contrast smaller-scale spatial variability of the prey fields between the two killer whale habitats (Table 1 and S1 Fig). All data were collected under the University of British Columbia Animal Care Permit no. A19-0053, Fisheries and Oceans Canada Marine Mammal Scientific License for Whale Research no. XMMS 6 2020, and United States Department of Commerce, NOAA, National Marine Fisheries Service Permit No. 23220.

**Table 1:** Summary of acoustic surveys conducted in the feeding habitats of northern (NRKW) and southern resident killer whales (SRKW).

Habitat	Survey areas	Total number of transects	Length of transects (mean ± SD; km)	Survey dates in 2020
NRKW	a	12	1.1 ± 0.3	Aug 25, 27
	b	7	1.1 ± 0.3	Aug 24
	c	17	1.8 ± 0.8	Aug 27
	d	8	2.9 ± 0.4	Aug 28
	e	9	1.7 ± 0.3	Aug 29
	f	22	1.7 ± 0.4	Aug 20,21
	g	11	0.9 ± 0.3	Aug 19
	h	15	1.0 ± 0.2	Aug 31, Sept 1
SRKW	a	10	5.3 ± 3.4	Sept 9, 10
	b	10	4.7 ± 1.3	Sept 8
	c	17	2.5 ± 1.1	Sept 6
	d	9	1.6 ± 0.7	Sept 5
	e	14	1.6 ± 0.5	Sept 5
	f	8	4.9 ± 1.0	Sept 14
	g	8	4.7 ± 1.2	Sept 15
	h	10	4.4 ± 1.7	Sept 13

Survey areas (a-h) correspond to the locations identified in Fig 1, and are alphabetically and geographically ordered from west to east. Tabulated data include sampling dates, numbers of transects within each surveyed area, and the average length of each transect.

We based our temporal survey window (Aug 19^th^ to Sept 15^th^ 2020) on expected returns of Chinook salmon, and long-term observations of killer whale presence [2, 15, 21, 22]. We accounted for interannual changes in southern resident killer whale use of the Salish Sea [23] by selecting time windows with consistently high likelihoods of whale presence to compare Chinook densities available to foraging northern and southern resident killer whales. We chose the first two weeks of September to sample the Salish Sea due to the consistence presence of southern residents during this month over past decades. We similarly chose the last two weeks of August to sample the North Island Waters when northern resident killer whales were expected to be present—and found both killer whale populations within both regions during our respective surveys. With only one research vessel, simultaneous sampling of both areas was not possible. Nor was it possible to do night surveys. We assumed daytime data were representative of nighttime conditions based on acoustic tracking of homeward bound adult Chinook, and that migration timings were similar whether fish arrived in the Salish Sea from the north or from the west. Thus, our study was designed to provide a snapshot of the densities of Chinook salmon available to resident killer whales targeting returning adult Chinook in hot-spot feeding areas during late summer.

We relied on fishery assessment data derived from the Albion test fishery to determine relative numbers Chinook salmon returning to the Fraser River between the months of April and October [24, 25]. To address the temporal offset between the migration timing of Chinook salmon detected by the Albion test fishery 50 km from the mouth of the Fraser River [26] with the timing of our surveys, we used recreational fishery assessment data [27] corresponding to Pacific Fishery Management Areas (PFMA) 12, 18, 19, 20, and 29. The recreational fishery assessment provides monthly angling catch estimates of both retained and released Chinook salmon in British Columbia waters [28]—and provided a qualitative measure of migration timings of Chinook salmon within each survey area.

Sampling was designed to characterize spatial variability of the prey field within the hot-spot feeding areas used by northern and southern resident killer whales. Within each feeding area we collected acoustic data using Simrad EK80 echosounders operating at 38 (10° split-beam), 70, 120, and 200 kHz (7° split-beams). The transducers were mounted on the starboard side of an 18-m vessel (MV Gikumi) at a depth of 0.9 m, with a maximum spatial overlap of the beams. The EK80 produced continuous wave signals with fast ramping applied, and operated at maximum ping rate (typically 0.5−1.4 pings·s^−1^) with a pulse duration of 512 μs and a vertical resolution of 9 cm—and was calibrated using a standard sphere method [29]. Vessel speed while conducting the acoustic surveys ranged from 4 to 6 knots (2.1−3.1 m·s^−1^).

We assessed the spatial variability of water properties between our two study areas by recording vertical conductivity–temperature–depth (CTD) profiles of temperature and conductivity (19plus V2 in 2019; Sea-Bird Electronics), oxygen (SBE 43; Sea-Bird Electronics), and fluorescence (WET Labs ECO). The profiles were collected along semi-randomly selected multi-frequency hydroacoustic transects, with measurements taken 5 m above the bottom depth. A total of 26 profiles were recorded (13 from each of the northern resident killer whale and southern resident killer whale regions). Instrument lags were corrected, and raw data was converted into variables of interest using factory calibrations.

### Data processing and analysis

We pre-processed the acoustic data using Echoview software (version 9.0; Echoview Software Pty Ltd.). To eliminate near-field transducer effects and surface backscatter bubbles, we excluded data shallower than 5.0 m depth from the analysis. Data within 2 m of the bottom were also removed from the analysis after visual inspection—and background noise was removed using a method developed by De Robertis and Higginbottom [30], with a minimum signal-to-noise ratio of 6 dB and a maximum noise threshold of −125 dB re 1 m^-1^. Target strength (TS; dB) and target range were computed using the average of all CTD down casts within each region, with sound speed [31] and absorption coefficients [32]. We also visually inspected our data to remove anomalies such as false bottoms and noise spikes.

To detect aggregations of fish (i.e., backscatter with defined, closed edges [33]), we employed the school detection module in Echoview with a threshold of −60 dB re m^-1^, and the detection criteria of Sato et al. [34]. Detected fish aggregations were excluded from further analysis to prevent misclassification of small fish aggregations as large individual targets.

We used Echoview software and employed a threshold of −28.5 dB at 38 kHz to isolate individual targets (i.e., large individual scatters at densities ≤1 per reverberation volume [35]) that could be potential killer whale prey comparable in size to an age-4 Chinook or larger (i.e., > 81 cm fork length). Although mean sizes-at-age of adult Chinook salmon have declined in the North Pacific since the 1970s by ~6% [36], they remain larger than the 81 cm detection cut off we used for age-4+ Chinook.

The frequency threshold we applied to the hydroacoustic data to detect age-4+ fish was established by Sato et al. [5] using empirical regressions formulated by Love [37] because no accurate dorsal aspect estimates of the target strength of adult salmon are currently available. The target strength value for a single target was calculated using two main inputs: (1) the mean fork lengths of Chinook salmon of different ages obtained from Ford and Ellis [2], and (2) the empirical equations for individual fish developed by Love [37]. Additionally, we calculated the average target strength over a range of ±45° to account for slight deviations in the natural orientations of the fish. A pulse length determination level was set to 12 dB, and normalized pulse lengths were between 0.8 and 2.0. The maximum beam compensation for transducer directivity correction was limited to 12 dB. To ensure that all scattering within the measured pulse length came from a single target, the maximum standard deviation of minor- and major-axis angles of the beam were set to 3°.

We computed the density of individual fish in a given water column as the number of fish per unit area. The sampling volume expands as the range increases when using the downward-facing transducer. In order to normalize fish density estimates for the larger sample volume, the detected fish were adjusted to represent a 1-m wide area at the surface using the following formula [38, 39]:

$$F_{w}=1/\left[2∙R∙\tan(3.5{}^{\circ})\right]$$ (1)

where *F*_w_ is weighted fish, *R* equals range, and 3.5° is one-half the nominal transducer beam width. For instance, at a depth of 8.2 m beneath the transducer, the cone-shaped detection area has a diameter of 1.0 m based on a 7° beam width. A fish detected at this depth is considered equivalent to one weighted fish at the surface, with measurements normalized to a 1-m transect width. The fish densities (ind.· m^−2^) for each transect were calculated by summing weighted fish by transect and dividing that by transect length.

The number and length of the line-transects we completed in each hot-spot area varied depending on the size of the area sampled. The areas had 7 to 22 transects of varying lengths (0.3–9.6 km; Table 1 and S1 Fig)—none of which could be simultaneously surveyed. Estimating the areal densities as the number of fish per unit area (ind.·1000 m^−2^) allowed us to obtain normalized densities independent of the lengths of the line-transects. We used boxplots and Mann–Whitney U tests to compare the areal densities between the two habitats.

To investigate how fish were distributed relative to the surface and bottom of the ocean, we plotted the depth at which all fish >81 cm occurred relative to the depth of the water column. We also split the data in two based on the depth of the water: shallow areas (< 50 m) and deep areas (> 50 m). We first analyzed the shallow water data, to understand the distribution and densities of Chinook salmon relative to the surface. We then analyzed the locations of fish in deep waters to understand how they were distributed relative to the bottom bathymetry. Statistical significance was assessed using *t*-tests (*p* < 0.05) to compare the mean distances of fish from the ocean bottom across different habitats.

## Results

### Timing of the field surveys

**Fig 2:**
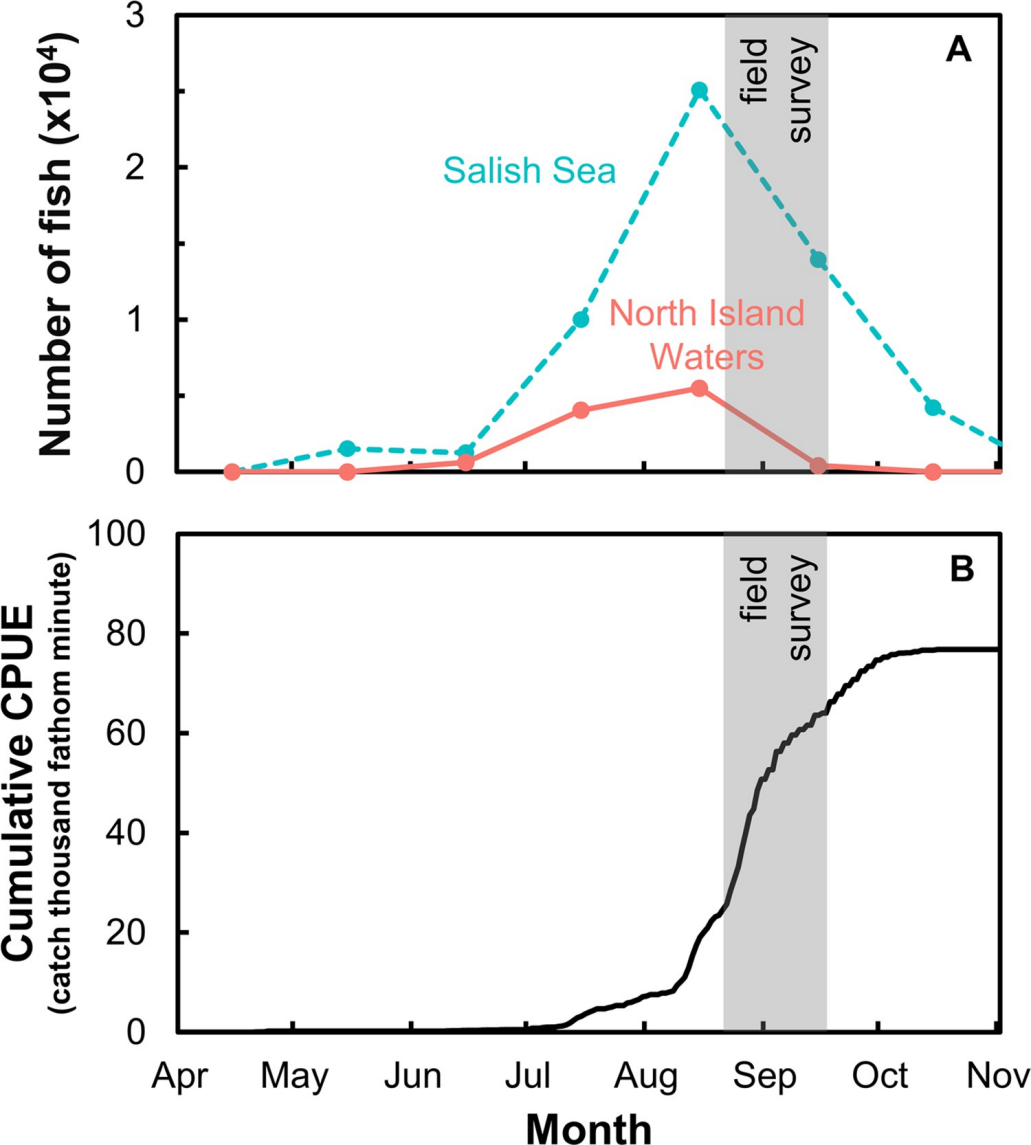
Timing of our surveys relative to the numbers of Chinook caught in the ocean by sport fisheries, and in the Fraser River by a test fishery. A) Numbers of Chinook salmon caught per month are based on released and retained individuals reported in the recreational fishery assessments conducted by Fisheries and Oceans Canada in the North Island Waters (red solid line) and Salish Sea (turquoise dashed line) in 2020. B) Relative cumulative abundance (catch per unit effort; CPUE; 1 fathom = 1.8 m) of Chinook salmon ∼50 km upstream from the mouth of the Fraser River as determined by the Albion test fishery. Gray bars represent the time window of our field survey. Note that some areas were closed in 2020 to recreational fisheries in Canada, and seasonal limits were placed on numbers that could be retained to protect declining Chinook stocks thought to be vital to the survival of southern resident killer whales. Thus, reduced catches do not necessarily reflect reduced abundance.

Our field surveys occurred as peak returns of Chinook salmon were passing through our survey areas, as determined by recreational catch estimates in 2020 (Fig 2A)—and by the numbers of Chinook salmon caught in the Albion test fishery near the mouth of the Fraser River (Fig 2B).

### Environmental conditions

In the North Island Waters where northern resident killer whales occurred, the temperature and salinity of the water remained relatively stable throughout the water column. Surface temperature was around 11°C, while the salinity ranged between 31–32 psu. At greater depths, the temperature dropped to 8–9°C, and the salinity increased slightly to 32–32.5 psu. In contrast, the vertical structure of the water column was more variable in the Salish Sea where southern resident killer whales occurred. Although deep water remained relatively stable (mean 143 m, 8.8°C, 32.2 psu, n = 11 stations), the upper layer water temperature ranged from 7–11.5°C, and the salinity varied between 27–33 psu. In addition, the fluorescence maxima occurred at < 25 m-depth, with values of 0.9 mg·m^-3^ in the North Island Waters, and 1.12 mg·m^-3^ in the areas sampled in the Salish Sea.

### Prey characteristics

**Fig 3:**
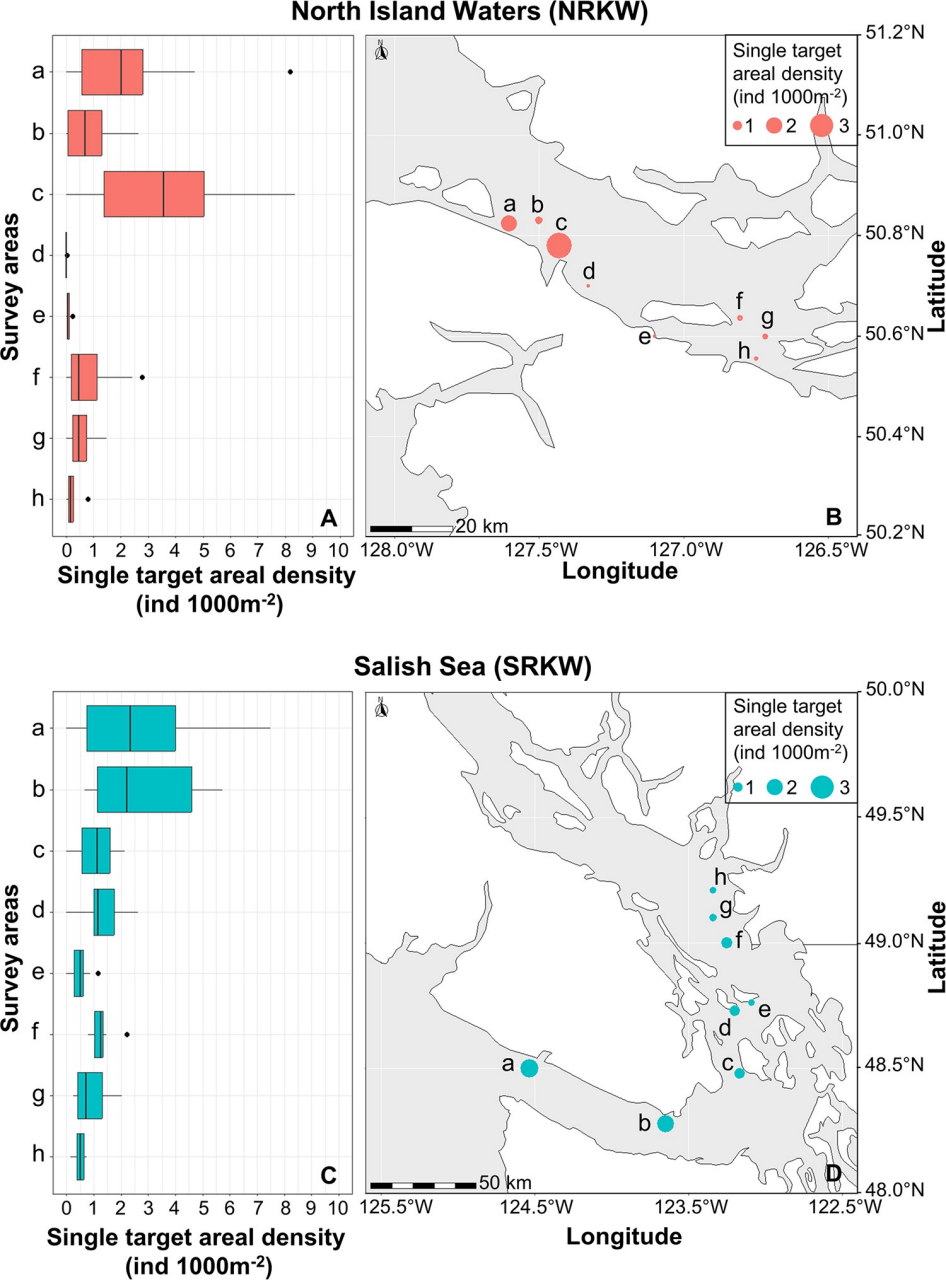
Areal densities of single targets in the feeding areas of northern and southern resident killer whales. The sizes of circles plotted on the maps are proportional to the densities of fish at each sampling location used by northern resident killer whales in the North Island Waters (A, B) and by southern resident killer whales in the Salish Sea (C, D). Bar graphs (A, C) show interquartile ranges of single target areal densities, and the circles on the maps (B, D) represent the medians. Prey densities differed significantly between feeding areas, and generally decreased as the fish migrated from west to east towards the Fraser River. Note that the areas surveyed (a–h) are ordered from west to east, and correspond to the directional movement of salmon returning to the Fraser River. Base maps were drawn using the R packages rnaturalearth and sf with free vector and raster map data from naturalearthdata.com.

Prey distributions were patchy and highly variable in both killer whale habitats—with concentrated areas of high fish densities occurring at both regional and local scales. Overall, the spatial distribution of prey was significantly heterogeneous across all study sites (Fig 3).

**Fig 4:**
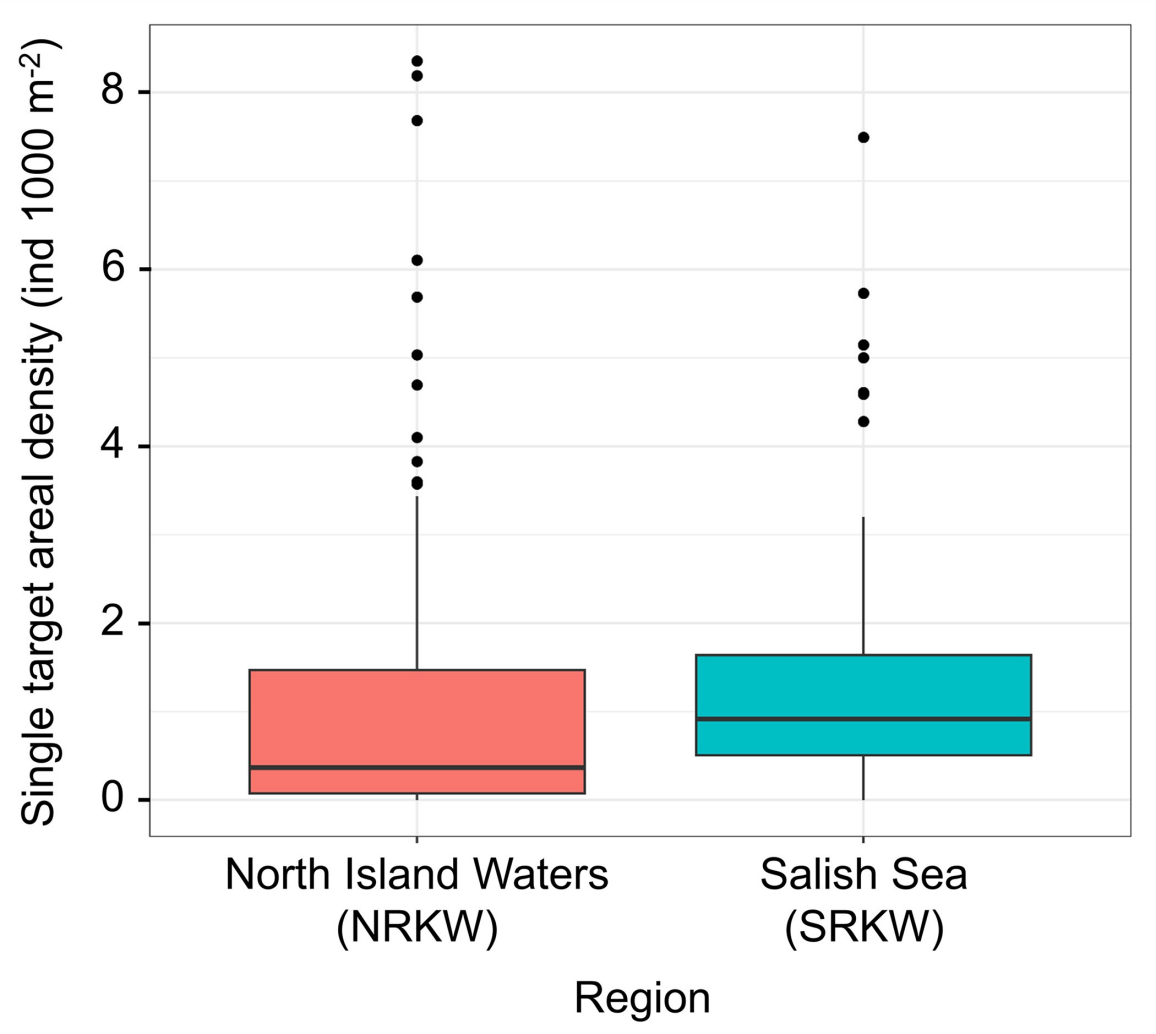
Comparison of areal densities of single targets in the hot-spot feeding areas used by resident killer whales in the North Island Waters and Salish Sea. Areal densities are the numbers of large fish present below a surface area of 1000 m^2^ in the feeding areas of northern resident killer whales (NRKW, North Island Waters, n = 101) and southern resident killer whales (SRKW, Salish Sea, n = 85). The box represents the 25^th^ and 75^th^ percentiles and the horizontal line shows median. The vertical lines (whiskers) extend from the box show the range of the data and dots indicate outliers. Prey densities differed significantly between northern and southern resident killer whale habitats (*p* < 0.05, Mann-Whitney *U* test).

The range of areal densities of Chinook in surveyed areas were similar in both habitats (0−3.4 ind.·1000 m^−2^ for northern resident killer whales, and 0−3.2 ind.·1000 m^−2^ for southern residents), but were more widely dispersed in northern resident habitat than in southern resident habitat (Fig 4). Typical areal densities (or the central tendency) was 0.9 ind.·1000 m^−2^ (median) in southern resident areas and 0.4 ind.·1000 m^−2^ (median) in northern resident habitat. However, some sampled areas had areal densities of 3–8 fish indicating a significantly patchy distribution of adult Chinook (Fig 4).

Areal densities of Chinook salmon were positively skewed in both habitats (Figs 3 and 4), particularly in the northern resident habitat where 50% of the areas surveyed had few Chinook. In contrast, there was a more even distribution of Chinook within the southern resident habitat suggesting a greater likelihood of encountering Chinook in all the areas surveyed within the southern resident habitat. Thus, the probability of encountering an individual Chinook was higher in the southern resident killer whale habitat than in the northern resident habitat (*p* < 0.05, Mann–Whitney *U* test). This implies that concentrations of salmon were higher in southern resident areas compared to northern resident areas.

**Fig 5:**
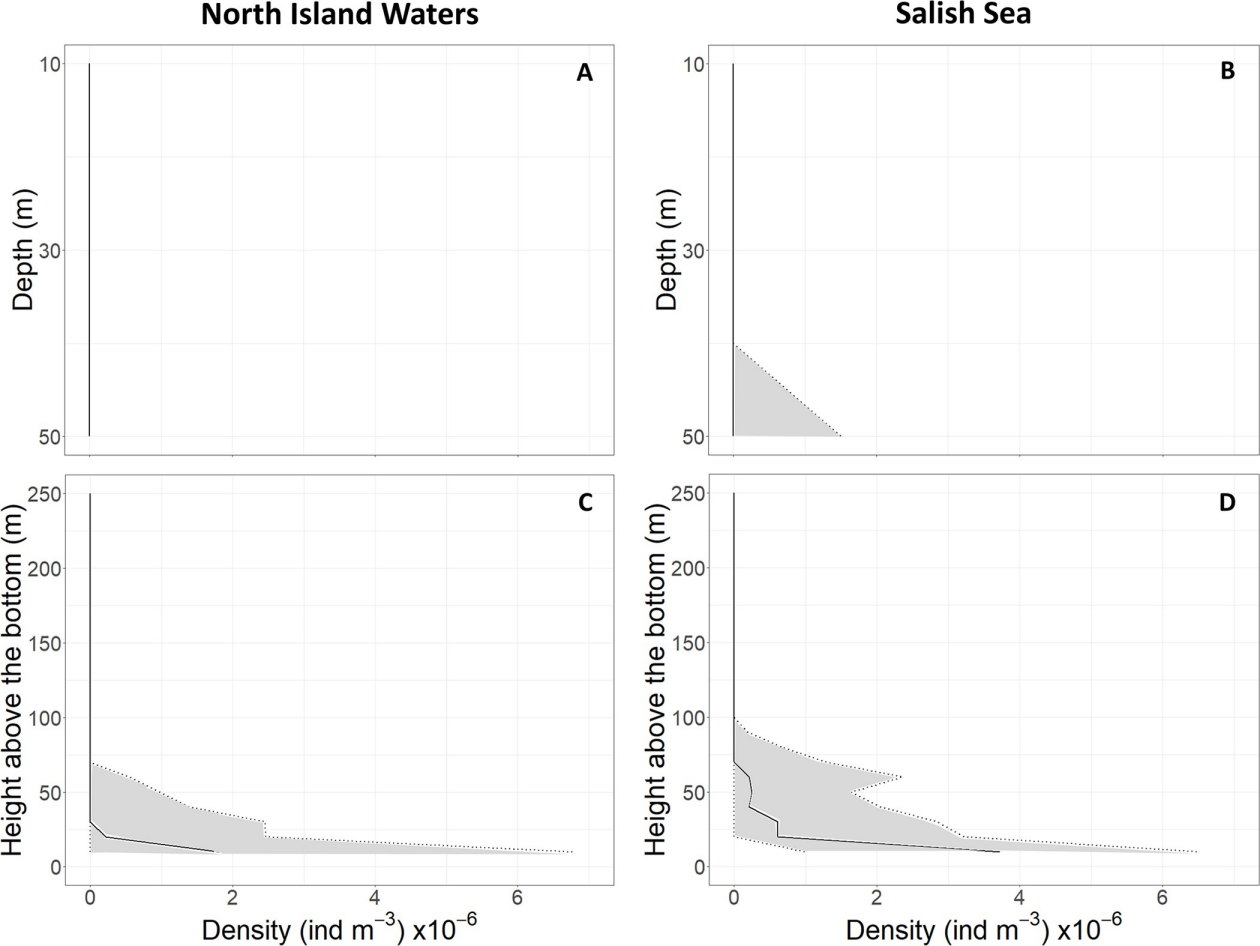
Densities of single targets near the surface and above the bottom of the hot-spot feeding areas sampled in the North Island Waters and Salish Sea. Densities per cubic meter of water are shown as a function of depth (0–50 m) below the surface (A, B) and as a function of distance (0–250 m) above the bottom where northern (C) and southern resident killer whales (D) feed. Solid lines show medians and dotted lines show 25th and 75th percentiles.

**Fig 6:**
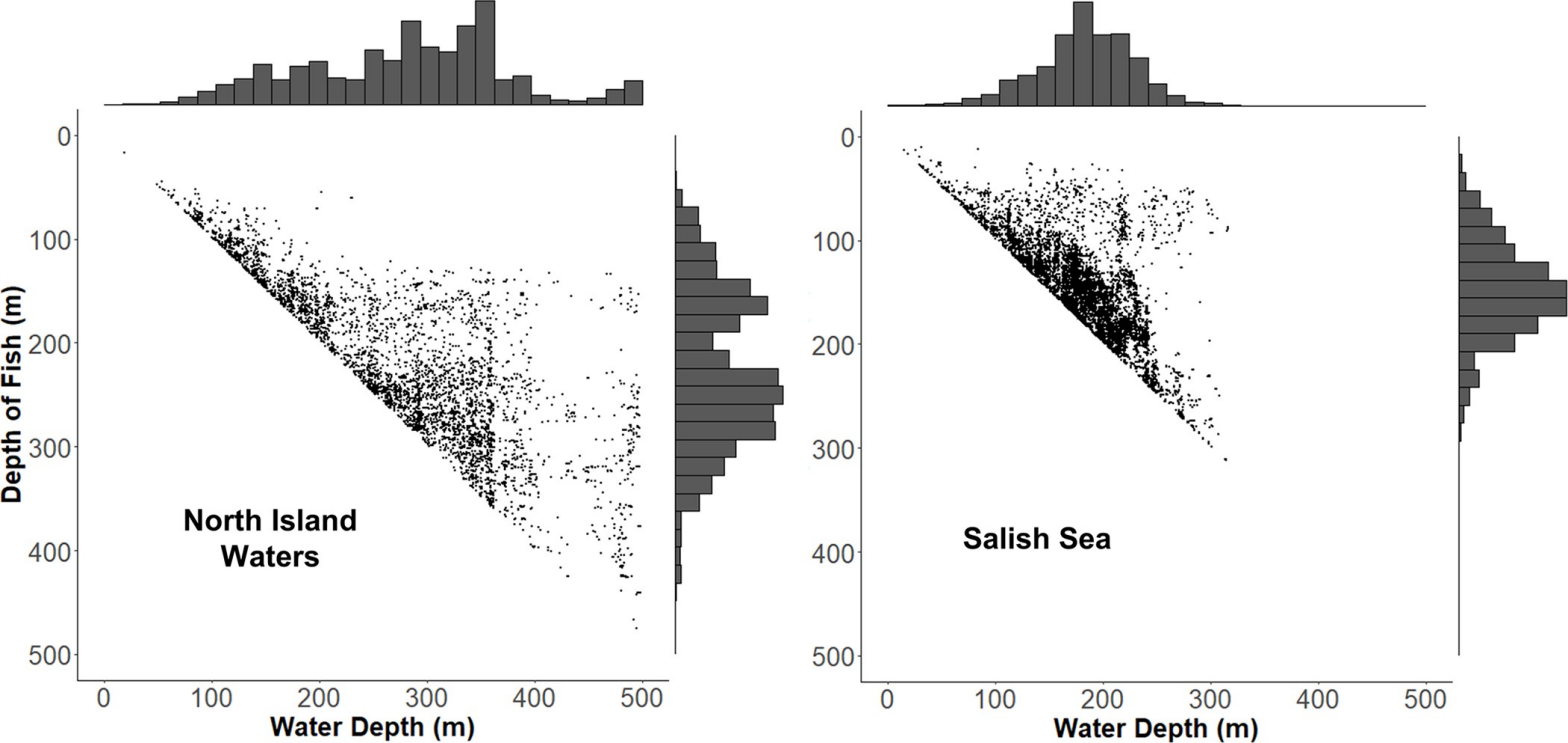
Depth distributions of Chinook salmon relative to water column depths where they occurred within 16 hot-spot feeding areas used by northern and southern resident killer whales. Each data point represents a fish >81 cm (n = 5,151 in the North Island Waters, and n = 9,109 in the Salish Sea) and shows the depth at which it occurred relative to the water column depth within the surveyed areas. Only fish occurring 2 m or more above the bottom are shown. All depths are plotted with a 1-m resolution.

In terms of the distribution of prey within the water column, big Chinook appeared to be largely absent or very few in numbers near the surface in both habitats (Figs 5A, 5B and 6). In waters > 50 m deep, prey predominantly occurred near the bottom (Figs 5C, 5D and 6). On average, single targets occurred 56 m from the ocean bottom in the North Island Water areas, and were 34 m on average above the bottom in southern resident killer whale areas. These differences were statistically significant (*t*-test; *p* < 0.05).

In summary, the majority of our transects in the North Island Waters surveys contained low prey densities, with a density of ≤ 1 ind.·1000 m^−2^ (with a few notable exceptions at Goletas Channel and Hardy Bay) (Fig 3A). In contrast, the prey densities in the Salish Sea areas were mostly > 1 ind.·1000 m^−2^ (with particularly high values observed in East Juan de Fuca and Port Renfrew) (Fig 3C). Another notable pattern within both study areas, was a general decrease in prey densities as fish migrated from west to east towards the Fraser River (Figs 3D and 4B).

## Discussion

We sought to assess the densities of large Chinook salmon available to northern and southern resident killer whales in 16 hot-spot feeding and fishing areas in the North Island Waters and Salish Sea of the Northeast Pacific to determine whether fewer salmon were available to the small population of southern residents compared to northern residents during summer. Counter to the hypothesis that there are fewer prey in the Salish Sea, we found no evidence that southern resident killer whales were limited by prey in the summer of 2020, compared to northern resident killer whales. Instead, median densities of prey were twice as high in southern resident killer whale habitat than in northern resident killer whale habitat.

### Densities of Chinook in killer whale habitats

Sato et al. [5] undertook a broad-scale survey in Johnstone Strait and Juan de Fuca Strait in 2018 and 2019, and found—as did we—that the distributions of Chinook salmon were patchy in both habitats. They also found the numbers of fish within each patch were 4–6 times higher in southern resident killer whale habitat than in northern resident killer whale habitat.

In contrast to Sato et al. [5], we undertook fine-scale surveys in 2020 of hot-spot areas traditionally used by sport fisheries and killer whales over a greater geographic range in the North Island Waters and Salish Sea. As such, we concentrated our surveys on near-shore areas and much smaller portions of Johnstone Strait and Juan de Fuca Strait than covered by Sato et al. [5]. Nevertheless, within the overlapping areas we sampled, we found Chinook densities tended to be higher in the southern habitat (e.g., median = 2.3 ind.·1000 m^−2^ at East Juan de Fuca and Port Renfrew vs. 0.3 ind.·1000 m^−2^ at Swanson Island, Johnstone Strait NW) (Figs 3 and 4). However, we found a general decrease in the areal densities of Chinook from west to east in both the Salish Sea and North Island Waters (Fig 3).

Densities in some surveyed areas had high standard deviations that occasionally exceeded the mean values. As such, we considered the median density of a region to be a better measure of central tendency than the mean to compare densities of salmon across different regions and surveys. We did not use the mode to investigate the probability of whales encountering large salmon as done by Sato et al. [5] because the mode failed to provide information about the distribution of low and high densities of salmon given the differences in the number and length of transects in our study. It was therefore better to use the median of the densities in each survey to compare densities between surveys.

Similar to Sato et al. [5], we found that the vertical depth distribution of Chinook in the southern resident killer whale habitat showed a preponderance of fish occurring near the bottom (between the bottom and ~25 m above the bottom in both studies—2018, 2019 and 2020) (Fig 5D). However, the second layer of concentrated Chinook occurred ~60 m above bottom, and was not subsurface (10–30 m) as reported by Sato et al. [5] (Fig 5B). In all likelihood, the subsurface concentration of Chinook detected by Sato and colleagues were individual fish searching for and following natal stream odors further from shore through Juan de Fuca Strait (which spans USA and Canadian waters)—while the hot-spot areas we sampled were located closer to shore where Chinook swimming in the upper water column would be more vulnerable to predators such as killer whales and sea lions.

A recent tracking study of acoustically tagged fish found most returning adult Chinook remain near the surface (< 30 m deep, mean 22 m), and rarely descend below 100 m while migrating through the Salish Sea [40]. However, there was a notable exception at San Juan Island, where an acoustic receiver placed within our study area (Fig 1B, Area c) detected fish throughout the water column to depths exceeding 100 m [40]. This suggests that the hot-spot feeding areas used by resident killer whales are likely places where Chinook encounter higher densities of forage fish, and where their risk of being caught by killer whales is also greatest.

The relatively shallow depths used by most of the acoustically-tagged migrating salmon [40] are remarkably similar to the depths where killer whales initiate echolocation to locate salmonids (<40 m) [41]. The shallow depths used by migrating Chinook are also consistent with the swimming patterns of resident killer whales that appear to make sustained foraging dives between 10–30 m, with very few dives deeper than 100 m [42]. Collectively, these findings suggest that resident killer whales typically search for Chinook in the upper water column (<30 m)—and pursue them to depths exceeding 100 m where captures have typically been recorded in the Salish Sea and North Island Waters [41, 43, 44]. However, this foraging strategy is unlikely to be as effective in areas where Chinook concentrate and are more vulnerable to capture—such as in the hot-spot areas we studied.

Our data on Chinook presence in hot-spot feeding areas align with the hypothesis that Chinook seek refuge in deeper waters where threats posed by killer whales and sea lions are highest. This high perceived risk of predation also likely explains the faster swimming speeds and reduced time spent by acoustically tagged Chinook in areas frequented by southern resident killer whales (unpublished data). As a result of altered Chinook behavior, killer whales would have to make more targeted, deeper dives to successfully capture prey in these specific regions.

In the North Island Waters, we found no indication of a bimodal vertical distribution of Chinook in 2020, which is consistent with the findings of Sato et al. [5] for 2018 and 2019. Similar to their study, we also found the Chinook were concentrated between the bottom and ~30 m above the bottom (Figs 5C and 6A)—with very few, if any Chinook, in shallow water (< 50 m) (Figs 5A and 6). This apparent absence of Chinook in shallow water may mean that the North Island Waters are an area of high predation risk.

### Patchy distributions and vertical movements of Chinook

Early tracking studies of Chinook salmon revealed that handled fish tended to exhibit escape responses after release, diving deep, with larger fish diving deeper than smaller ones [45]. Subsequent tracking studies have corroborated these findings, indicating that Chinook salmon swim deeper and move slower compared to other salmon species [46, 47]. However, movement studies have also revealed that adult Chinook mostly stay within 30 m of the surface, and make infrequent use of deeper depths (> 100 m), as they migrate towards their natal rivers [40]. Such complex vertical movements and distribution patterns are believed to reflect seasonal and daily differences in foraging effort, predator avoidance, and navigational needs—which are in turn a function of bathymetry, maturation stage, and spatial locations where they occur [48–50].

Considerable attention has been given to the possible effects that predation can have on the numbers, body sizes and age at maturation of Chinook salmon [51, 52]. However, little consideration has been given to the role that predation plays in shaping the distribution and behavior of Chinook salmon.

Chinook face predation risks at each stage of their life cycle, which influences their feeding and migration patterns as they seek to avoid areas with high predator activity [53–55]. Thus, we believe that the predation pressure imposed by killer whales and other predators can account for the deep depths and low spatial densities of the Chinook we noted in the hot-spot areas where killer whales feed. Similarly, predation pressure by killer whales may explain the extreme patchiness of Chinook relative to other salmon species (i.e., why only a few of the hot-spots we sampled had densities of 3–8 fish, while most surveyed areas had median densities of just 0.4–0.9 ind.·1000 m^−2^). The extreme patchiness of Chinook suggests they may enhance their survival chances by migrating in small, scattered groups rather than in large, dense schools.

Prey patchiness (the aggregation of individuals) is known to significantly shape predator-prey interactions [56–58]. Predators that focus their foraging in areas where prey densities tend to be higher may cause prey to seek refuge or aggregate in patches to reduce individual predation risk. As such, prey patchiness is a significant determinant of foraging success, and directly impacts predator foraging strategies and prey population dynamics [59, 60]. Obtaining a better understanding of the causes and consequences of predation risk and prey patchiness relative to killer whales and Chinook salmon would contribute to better informing conservation efforts and ensuring the long-term availability of Chinook salmon for foraging killer whales.

### Implications for management and conservation

Although our fine-scale acoustic survey of Chinook salmon in 16 hot-spot feeding and fishing areas revealed greater densities of Chinook salmon in southern resident killer whale habitat (Salish Sea) compared to northern resident killer whale habitat (North Island Waters) during summer, it does not necessarily mean that the availability of Chinook was greater for killer whales in the Salish Sea. Availability is a function of abundance and accessibility of prey (i.e., the amount of Chinook present and the ease of obtaining it) [61]. Thus, while Chinook abundance was greater in southern resident killer whale habitat, it could be less accessible to them if acoustic and physical disturbance from vessels and other sources impede their ability to forage.

Studies of southern resident killer whales carrying suction cupped sound and movement tags have found the probability of killer whales capturing prey increases as salmon abundance increases, but is negatively affected by the movements and noise of nearby vessels [62, 63]. Vessel noise can mask communication between pod members and interfere with foraging and navigation [64, 65]—while the physical presence of vessels can reduce the foraging effort of resident killer whales [63, 66]. Killer whales are more likely to encounter greater numbers of vessels in the Salish Sea than in the North Island Waters, which could mean that salmon are less accessible to southern residents than to northern residents despite there being a higher abundance of Chinook.

All of the sites we sampled have been historically used by resident killer whales and sport fishermen—and are believed to be hotspots where densities of Chinook should be higher than unfrequented areas. However, median densities were not consistently high, and varied considerably between sites (ranging from 0.0–3.6 ind.·1000 m^−2^)—with an overall median density of 1.2 ind.·1000 m^−2^ for all hot-spot areas and regions combined at any given time. We encountered relatively few if any vessels fishing due to fishery closures—except West of San Juan Island where substantial fishing effort by recreational vessels likely lowered the density of fish present at the time of our survey (1.1 ind.·1000 m^−2^). Most of the fish we detected in all 16 areas were deep and near the bottom, which is consistent with depths where resident killer whales typically capture their prey [41, 43, 44].

Average depths of areas we sampled were 128 m in the North Island Waters and 139 m in Salish Sea, which corresponds to maximum reported dive depths associated with fish captures of 91 m for northern resident killer whales, and 109 m for southern resident killer whales [44]. This apparent difference in capture depths of northern and southern resident killer whales may be explained by relative differences in the bathymetries of the two regions, and the propensity of Chinook to seek being near the bottom in locations where they need to minimize their risk of being preyed on by sea lions and killer whales. This is notable as the Chinook we found (Fig 6) were typically deeper than the average depths of the surveyed areas, and tended to be more numerous in the deepest portions of them. Specifically, in the Salish Sea, most fish occurred at depths of roughly 100–200 m in waters of 160–240 m, while in the North Island Waters, they were more typically found at depths of 100–300 m in waters of 260–360 m (Fig 6). Thus, the Salish Sea had a higher abundance of fish at the dive depths commonly associated with killer whale captures of Chinook salmon compared to the North Island Waters.

The densities of salmon we observed tended to be highest in the western parts of the North Island Waters and Salish Sea, and became progressively lower as fish migrated eastward towards Johnstone Strait, or eastward towards the Strait of Georgia. Whether or not this drop in numbers reflected sequential predation, or possibly a difference in Chinook holding times and migration speeds, is unknown. Similarly, it is not clear how the numbers of fish that ultimately arrived at the Fraser River differed depending on whether they migrated via Johnstone Strait or through Juan de Fuca Strait.

During our study period, notably more southern resident killer whales spent time feeding near the entrance of Juan de Fuca Strait where we found the highest densities of Chinook occurred—and the whales conversely spent less time near the Fraser River where prey densities were lower. In contrast, the northern resident killer whales we encountered during our study appeared to be more evenly distributed in the North Islands Waters. In all likelihood, the Chinook we detected with echosounders were not resident fish, but rather migratory fish returning to their natal rivers in the Salish Sea. This is consistent with Chinook densities being fluid and not static due to differences in the sizes and timings of returns of different Chinook runs [67, 68].

Chinook remain in coastal waters for longer periods than other salmon species [69] with estimated mean migration speeds ranging from 7 to 66 km per day [40, 50]. However, Chinook occur in much lower numbers per unit area compared to other salmonids such as pink and sockeye salmon. Median densities of adult Chinook in prime killer whale foraging habitats were just 0.4 fish in northern resident killer whale habitat and 0.9 per 1000 m^2^ in southern resident habitat. How this compares to historic densities of Chinook is unknown. Nor is it yet known what optimum densities are needed to support killer whales and spawning Chinook populations.

Although not all foraging areas may have Chinook present at any given time, as shown by Fig 3, there is likely a minimum density threshold of fish—at least 0.4–0.9 individuals per 1000 m^2^ based on our findings—that must be encountered within a given time to make it energetically worthwhile for a whale to search its usual feeding spots. However, in terms of what ultimately matters to killer whales, it is not known how they respond to the high variability in prey densities between sites, nor whether they benefit more from encountering higher densities of Chinook in one or two areas rather than lower densities across all areas.

Ultimately, the foraging success of killer whales depends on the spatial and seasonal densities of Chinook salmon available to them. It is also affected by interannual differences in run sizes of salmon, and by the serial depletion of salmon numbers that occurs during migration (Fig 3). Such considerations highlight the spatial and temporal complexities facing resource managers that seek to ensure there are sufficient Chinook salmon available to support healthy numbers of killer whales. A better understanding of the behavioral ecology of the different types and runs of Chinook salmon is needed to guide effective management and conservation decisions designed to mitigate fishery impacts and protect populations of salmon and killer whales. This is particularly important in the face of climate change and other environmental stressors.

Our findings do not negate the hypothesis that southern residents are thinner on average than northern resident killer whales because they are not consuming enough prey [70]. Rather, they suggest that southern resident killer whales are no less food-limited than northern resident killer whales during summer, and any food shortage that might be occurring is happening elsewhere or at other times of the year. The Salish Sea, which is located at the northern boundary of southern resident killer whale habitat, comprises a small portion of their overall range (which includes all of the coastal waters between southern British Columbia and central California) [3, 22, 71]. Thus, our assessments of Chinook densities in the Salish Sea in 2020, along with those from 2018–2019 [5], suggest that southern resident killer whales are not food-limited in the Salish Sea—and that their low numbers and apparent low carrying capacity reflects lower prey abundances further south in Oregon or California.

Southern resident killer whales have traditionally been most active in the Salish Sea during the summer and autumn months [72] where most research and management efforts have been concentrated to date. Our research points to the need for a comprehensive assessment of the diets and availability of prey elsewhere in the range of southern resident killer whales along the coasts of Oregon and California during winter and spring when their presence in the Salish Sea has been historically sparse. Prey availability should also be assessed during winter months in the Salish Sea because of its relatively high use by the southern residents during winter (Nov and Dec). Whether or not their winter presence in the Salish Sea [72] reflects poorer conditions elsewhere or greater abundance of prey than in the past is unknown.

### Study limitations

Long-term continuous measurements are necessary to quantify intra-seasonal variability in the availability of Chinook by time of year to establish a more complete understanding of the dynamics of predator-prey interactions between Chinook salmon and resident killer whales. Sampling hot-spot feeding areas just once, as we have done, runs the risk of mistakenly assuming that the conditions under which we collected abundance estimates are representative of periods before and after our sampling windows.

Ideally, we would have had funds to increase our survey effort to do repeat transects under different tide states over entire summers and across multiple years to fully capture the heterogeneity of salmon movements, diversion rates, and interannual differences in relative abundances of different Chinook stocks. We suspect, however, that none of this augmented survey effort would alter our fundamental conclusion that the preponderance of Chinook salmon is greater in the Salish Sea than it is in the North Island Waters during summer as concluded from hydroacoustic surveys [5] and from catch-per-unit effort data obtained from recreational and commercial fisheries [67].

We tried to minimize error associated with variable run timings by designing our study to collect data when maximum numbers of Chinook were expected to move through the North Island Waters and Salish Sea towards the Fraser River and Puget Sound. Retrospective analysis of recreational and test fishery data shows our sampling occurred as peak numbers of returning fish began to subside (Fig 2). Resident killer whales were also present and feeding in both regions during our study, although they were not in the hot-spot areas while we surveyed, further supporting the appropriateness of our chosen sampling times. All of our sampling sites were located along the migratory pathways used by Chinook salmon [73]—and were selected because they are sites where killer whales are known to feed, and where sport and commercial fisheries have historically targeted Chinook.

In terms of our confidence in identifying Chinook salmon from acoustic back-scatter, we used the same technique and methods developed and validated by Sato et al. [5] who integrated complementary trawl and hook-and-line fishing to identify species and determine size composition and biomass. Unfortunately, we could not fish from our research vessel, which limits our confidence in differentiating the biomass of the smaller species of fish of different length distributions with overlapping backscattering characteristics.

It is possible that the target strength of wild fish we assigned to age-4+ Chinook could have been from species that rarely grow to such lengths in our survey areas [74]. However, some of the species that can attain such sizes (e.g., Pacific spiny dogfish, *Squalus suckleyi*) have lower target strengths than those of age-4 Chinook salmon [5]. Similarly, other fish species that have swim bladders and can attain large sizes (e.g., Pacific hake, *Merluccius productus* and walleye pollock, *Gadus chalcogrammus*) were easily excluded from the acoustic data using a school detection algorithm to exclude species that form large aggregations. We also excluded data within 2 m of the seafloor to remove species such as halibut that are linked to bathymetric features and the benthic environment. The only other large fish species that might have been missed is Pacific cod (*Gadus macrocephalus*), which primarily resides in deep waters near the seafloor, and would have likely been excluded from our analysis of near-bottom data. Considering the typical sizes of the fishes in the study areas, the exclusion of bottom-associated fish, and the predominance of large Chinook salmon caught through the trolling in the previous years, we can conclude that the large individual targets mostly consisted of large Chinook salmon.

Target strength is a key piece of information needed to accurately assess fish populations with acoustics [75]. Unfortunately, there is no dorsal-aspect target strength model available for Chinook salmon, which complicates using downward-looking echosounders. Previous studies on target strength have only considered side- and ventral-aspects of adult salmon [76–78]—and there is limited information available for dorsal aspect target strength of small kokanee salmon (*Oncorhynchus nerka*) [79]. However, there is no information available for the dorsal target strength of Chinook salmon other than this single frequency (which is not commonly used) and modeling estimates, as well as simulated broadband in-situ measurements [80–82]. Thus, we used the threshold of target strength (-28.5 dB) derived by Sato et al. [5] using empirical regressions [37] to estimate the density and sizes of > age-4 Chinook. In-situ measurements are needed to verify the accuracy of the empirical calculations of target strengths of adult Chinook salmon [83, 84].

## Conclusions

Understanding the spatial distributions and movements of Chinook salmon relative to the feeding ecology of killer whales is needed to resolve whether fisheries or other factors have negatively affected the availability of killer whale prey. Acoustic surveys are a powerful means to assess the abundance, distribution, and behavior of fish in near-real time [19], enabling researchers and managers to better understand the ecology of Chinook salmon and their interactions with predators such as resident killer whales. They can provide insights into the spatial distribution of Chinook salmon and how their distribution changes over time, which can in turn be used to guide management strategies that protect and enhance critical habitats for Chinook salmon and their predators.

Our fine-scale hydroacoustic surveys were designed to better understand the spatial variability of Chinook salmon within hotspot feeding areas used by resident killer whales during late summer—and to test whether there were fewer Chinook available to southern resident killer whales in the Salish Sea compared to northern resident killer whales feeding in the North Islands Waters. Contrary to expectations, we found that the areal densities of prey (large salmon) available to killer whales were higher in the southern habitat. Our findings are consistent with those of Sato et al. [5], and suggest that southern resident killer whales are no more food limited during late summer than northern resident killer whales. This implies that the difference in growth rates of the two populations is either due to other factors (e.g., inbreeding, disturbance, contaminants, competition) or that southern resident killer whales are experiencing a food shortage beyond the Salish Sea during winter or spring.

## Supporting information

S1 Fig Distribution of the transect lines used to acoustically assess the presence of Chinook salmon in hot-spot feeding areas used by southern (SRKW) and northern (NRKW) resident killer whales in 2020. Additional details about the transects within the survey areas (labelled a–h) of each region are contained in Table 1. Base maps were drawn using the R packages rnaturalearth and sf with free vector and raster map data from naturalearthdata.com.
(TIF)
