# Supplementary figures and images for: Prevalence of Chinook salmon is higher for southern than for northern resident killer whales in summer hot-spot feeding areas
Source: PLoS One. 2024 Oct 10;19(10):e0311388. doi: 10.1371/journal.pone.0311388 (PMC11469597; doi:10.1371/journal.pone.0311388)

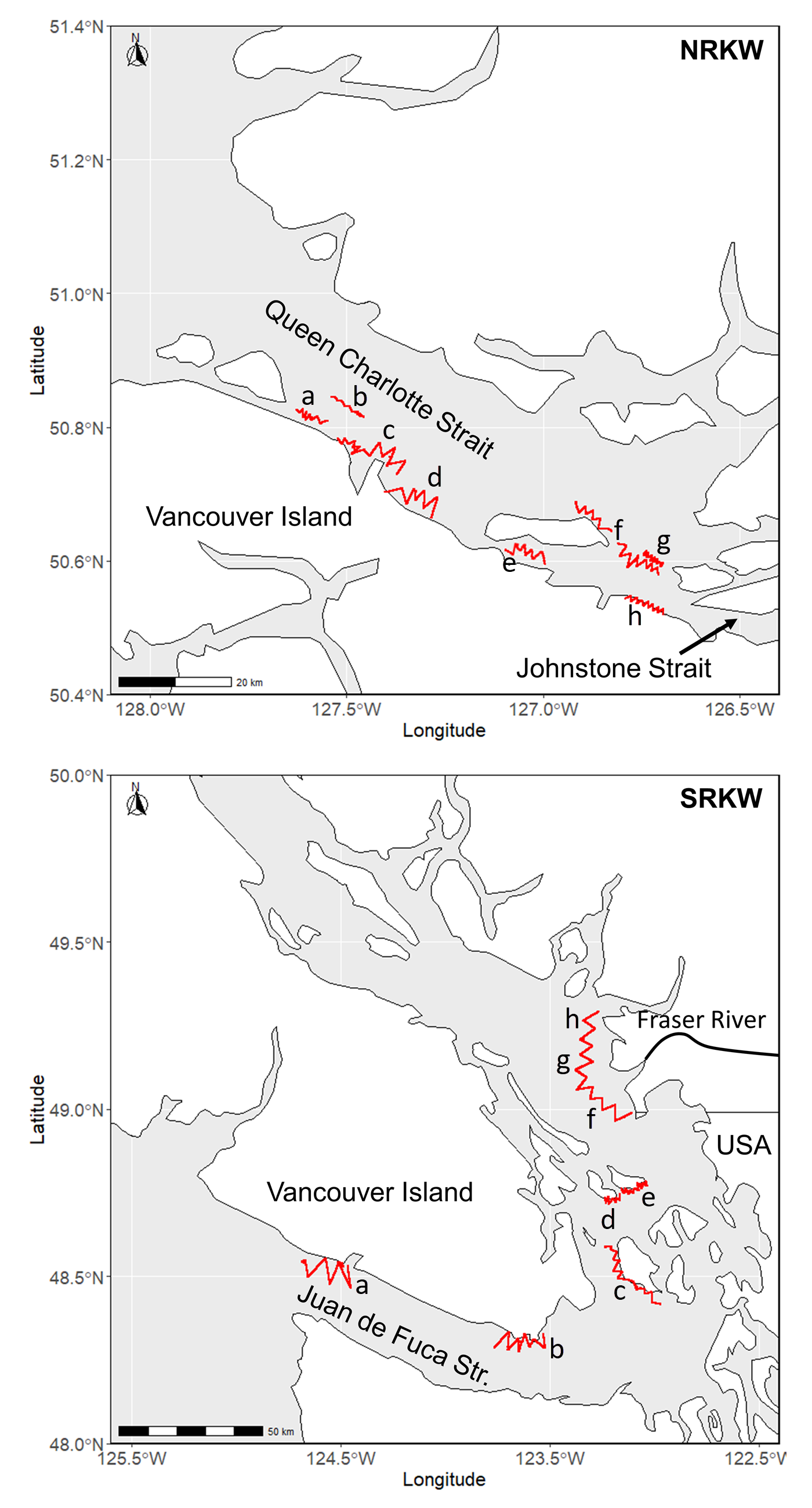

Supplement: S1 Fig — Additional details about the transects within the survey areas (labelled a–h) of each region are contained in Table 1. Base maps were drawn using the R packages rnaturalearth and sf with free vector and raster map data from naturalearthdata.com. (TIF) [file pone.0311388.s001.tif]
